# Alternative Therapy of Psychosis: Potential Phytochemicals and Drug Targets in the Management of Schizophrenia

**DOI:** 10.3389/fphar.2022.895668

**Published:** 2022-05-17

**Authors:** Ammara Saleem, Qurat-ul-Ain, Muhammad Furqan Akhtar

**Affiliations:** ^1^ Department of Pharmacology, Faculty of Pharmaceutical Sciences, Government College University Faisalabad, Faisalabad, Pakistan; ^2^ Riphah Institute of Pharmaceutical Sciences, Riphah International University, Lahore, Pakistan

**Keywords:** schizophrenia, phytochemicals, oxidative stress, flavonoids, dopamine

## Abstract

Schizophrenia is a chronic mental and behavioral disorder characterized by clusters of symptoms including hallucinations, delusions, disorganized thoughts and social withdrawal. It is mainly contributed by defects in dopamine, glutamate, cholinergic and serotonergic pathways, genetic and environmental factors, prenatal infections, oxidative stress, immune system activation and inflammation. Management of schizophrenia is usually carried out with typical and atypical antipsychotics, but it yields modest benefits with a diversity of side effects. Therefore, the current study was designed to determine the phytochemicals as new drug candidates for treatment and management of schizophrenia. These phytochemicals alter and affect neurotransmission, cell signaling pathways, endocannabinoid receptors, neuro-inflammation, activation of immune system and status of oxidative stress. Phytochemicals exhibiting anti-schizophrenic activity are mostly flavonoids, polyphenols, alkaloids, terpenoids, terpenes, polypropanoids, lactones and glycosides. However, well-designed clinical trials are consequently required to investigate potential protective effect and therapeutic benefits of these phytochemicals against schizophrenia.

## Introduction

Schizophrenia is a major debilitating disease of adults in every society, affecting about 1–1.5% of global population (Howes and Murray, 2014). The incidence of schizophrenia is higher among males than female at a ratio of 1.4 to1.0 (McGrath et al., 2008). Schizophrenia is the seventh most costly disorders in the world (Ross and Margolis, 2005; McGrath et al., 2008). It is a syndrome involving positive and negative symptoms, and cognitive problems (McCutcheon et al., 2019). Positive symptoms, including hallucinations and delusions are the foremost feature of this syndrome. Negative symptoms include the failure to express emotions and apathy. Cognitive problems arise before the appearance of psychosis and can act as better predictor of the disease (Dienel and Lewis, 2019). Unlike other degenerative diseases, its onset occurs during early adulthood or late adolescence (An der Heiden and Häfner, 2000). Schizophrenia predominantly occurs during second and third decade of the life, but it can also affect elderly individuals (Niemi et al., 2003). It increases the risk of other brain disorders such as Parkinson’s disease, autism, Alzheimer’s disease and multiple sclerosis (Brown, 2012).

A complex interaction of genetic, nutritional, microbial and environmental factors contribute to schizophrenia (Eyles, 2021). Several neurotransmitters such as Dopamine, gamma aminobutyric acid (GABA) and glutamate, serotonin and noradrenaline play significant role in the pathogenesis as well as progression of schizophrenia (Prestwood et al., 2021). Moreover, schizophrenia also results from interplay of neuro-inflammation, oxidative stress, cell signaling pathways and abnormal immune system activation with schizophrenia (Kokkinou et al., 2021).

Typical anti-psychotic drugs show higher affinity, stronger binding and more inhibition of dopamine receptors than the atypical anti-psychotic drugs. However, atypical anti-psychotic drugs are more effective than the typical antipsychotics due to their action at dopamine, serotonin and cholinergic receptors. Individual anti-schizophrenic drugs have variable efficacy in different patients (Bahta et al., 2021). Atypical antipsychotics are generally more effective, but have fewer side effects as compared to typical anti-psychotic drugs. General adverse effects of these synthetic drugs include but not limited to hormonal disturbances, vertigo, tardive dyskinesia, obesity, infertility, neuroleptic malignant syndrome, sedation and agitation. For avoiding these drug related problems, there is a great need of more efficacious and safer remedies (Prestwood et al., 2021).

Phytochemicals are of natural origin that offer cost effective, accessible and valuable source of drugs. Herbal therapies have played their beneficial role throughout human history. Humanity is turning towards herbal therapies due to questionable efficacy and toxic health implications of already used pharmacotherapy of schizophrenia (Datta et al., 2021). Moreover, the progress in developing synthetic anti-schizophrenic drugs is still glaringly slow because of diverse factors such as heterogeneity of schizophrenia phenotypes, ambiguous pathophysiology, pathological lesions, complex genetics changes and other risk factors (Yadav, 2021). Therefore, the phytochemicals offer potential and diverse alternatives to allopathic anti-schizophrenic medicines due to their wide array of biological activities such as anti-inflammatory activity, anti-oxidant potential, affecting neurotransmission, and modulating cell signaling pathways (Arnold et al., 2005; Ross and Margolis, 2005; Rapoport et al., 2005)

Pharmacotherapy of schizophrenia is usually carried out with typical and atypical antipsychotics, but these drugs yield only modest benefits with a diversity of side effects. Phytochemicals are diverse chemicals that offer themselves as useful alternative to conventional allopathic treatments. Therefore, the current review was designed to determine the potential of anti-schizophrenic phytochemicals as new drug candidates and, the pre-clinical and clinical progress regarding their antipsychotic action.

### Risk Factors of Schizophrenia

Schizophrenia is a complex disease which remains rudimentary and has involvement of various genetic, nutritional, microbial and environmental factors (Caspi and Moffitt, 2006; Lewis and Sweet, 2009). A person can have several defective genes, but risk factors such as infections, drug abuse and obstetric complications may conclusively lead to illness (Craddock et al., 2005; Winterer et al., 2003). Infections like influenza, rubella, cytomegalovirus, *Toxoplasma gondii*, herpes simplex virus 1 and 2, and polio virus can predispose the vulnerable individuals to schizophrenia (Brown and Susser, 2002). Obstetric complications including low birth weight, premature birth, rhesus incompatibility, resuscitation at birth time, nutritional deficiency of fetus and emergency caesarean delivery have been strongly correlated to the disease (Kyle and Pichard, 2006; St Clair et al., 2005). After maternal infection, there is an increased production of cytokines that adversely affects the immune system culminating in brain damage (Brown and Derkits, 2010). Nutritional factors that can contribute to schizophrenia include continuous intake of high fat and high sugar diets, and deficiency of vitamin D, B9 and B12. Recent studies showed that a high level of maternal IL-8 had caused anatomical problems in fetus (Meyer 2011; Na et al., 2014).

**FIGURE 1:**
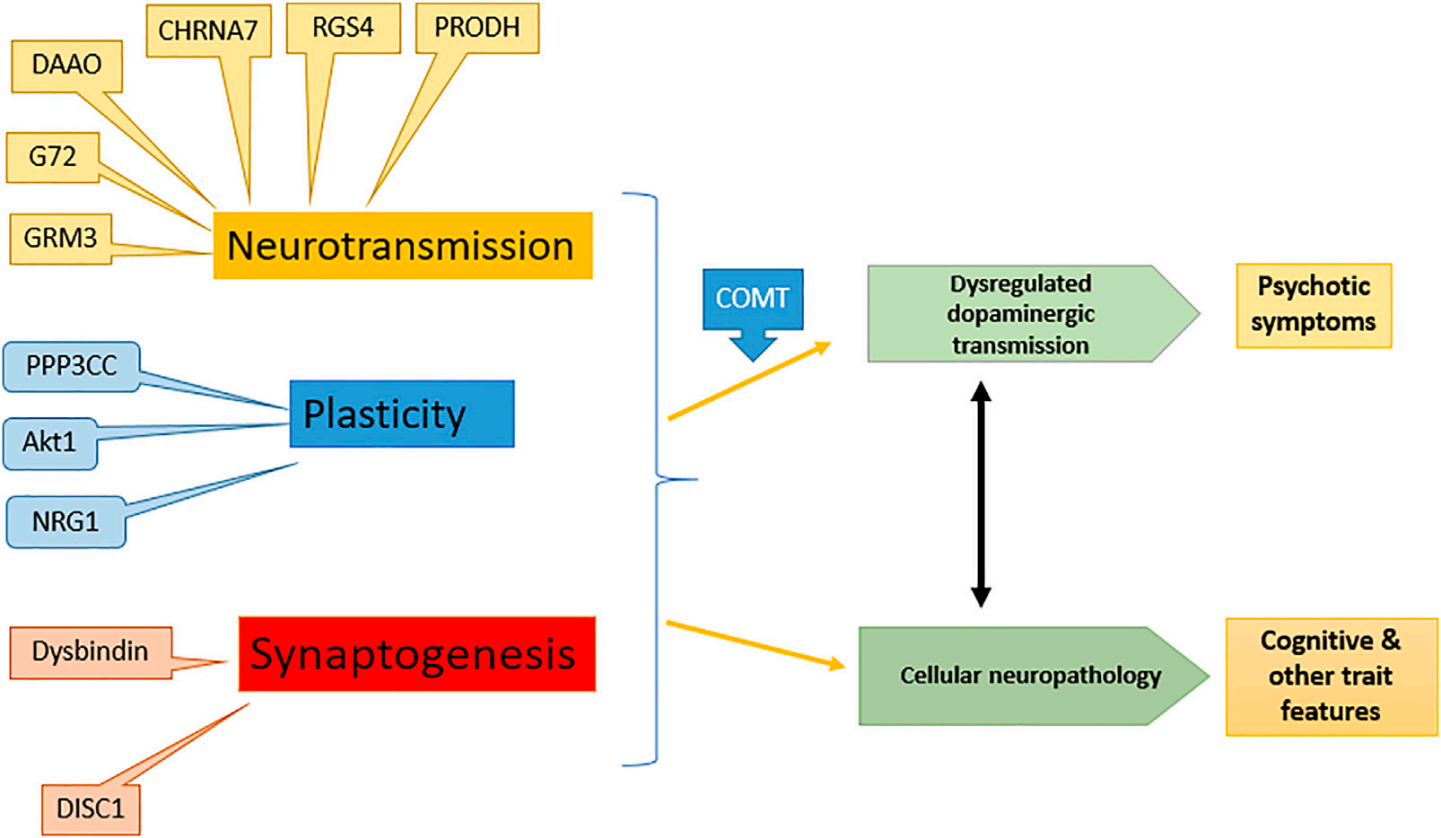
Effect of different genes on symptoms of schizophrenia: modified from (Harrison and Weinberger 2005). NRG1, Neuregulin-1; DTNBP1, Dystrobrevin-binding protein 1; DISC1, Disrupted in schizophrenia; DAAO, d-amino acid oxidase; RGSR, Regulator of G protein signaling-4; COMT, Catechol-O-methyl transferase; PRODH, Proline dehydrogenase, G72, d-amino acid oxidase activator.

Schizophrenia has a strong hereditary tendency, showing 10% chance in close relatives of patient. A complex interaction of one or more of 20 genes is responsible for the disease (Harrison and Weinberger, 2005). The genes including neuregulin-1 (NRG1), dysbindin (DTNBP1), disrupted in schizophrenia (DISC1), d-amino acid oxidase (DAAO), regulator of G protein signaling-4 (RGSR), catechol-O-methyl transferase (COMT), proline dehydrogenase (PRODH) and G72 are schizophrenia susceptible while several genes affect the glutamatergic transmission pathway in the brain (Craddock et al., 2005; Ross and Margolis, 2005). The effect of various gene expressions on schizophrenia is shown in Figure 1.

There is another phenomenon called endo-phenotypes that is responsible for different clinical symptoms e.g. cognitive defects, neurological abnormalities, impaired emotions and eye movement abnormality (Cannon, 2005; Ross and Margolis, 2005). Different genes control different endo-phenotypes of specific characteristics, inherited in a Mendelian fashion and can cause full schizophrenia if all genes are inherited together (Rapoport et al., 2005; Khandaker et al., 2015).

### Pathophysiology of Schizophrenia

**FIGURE 2:**
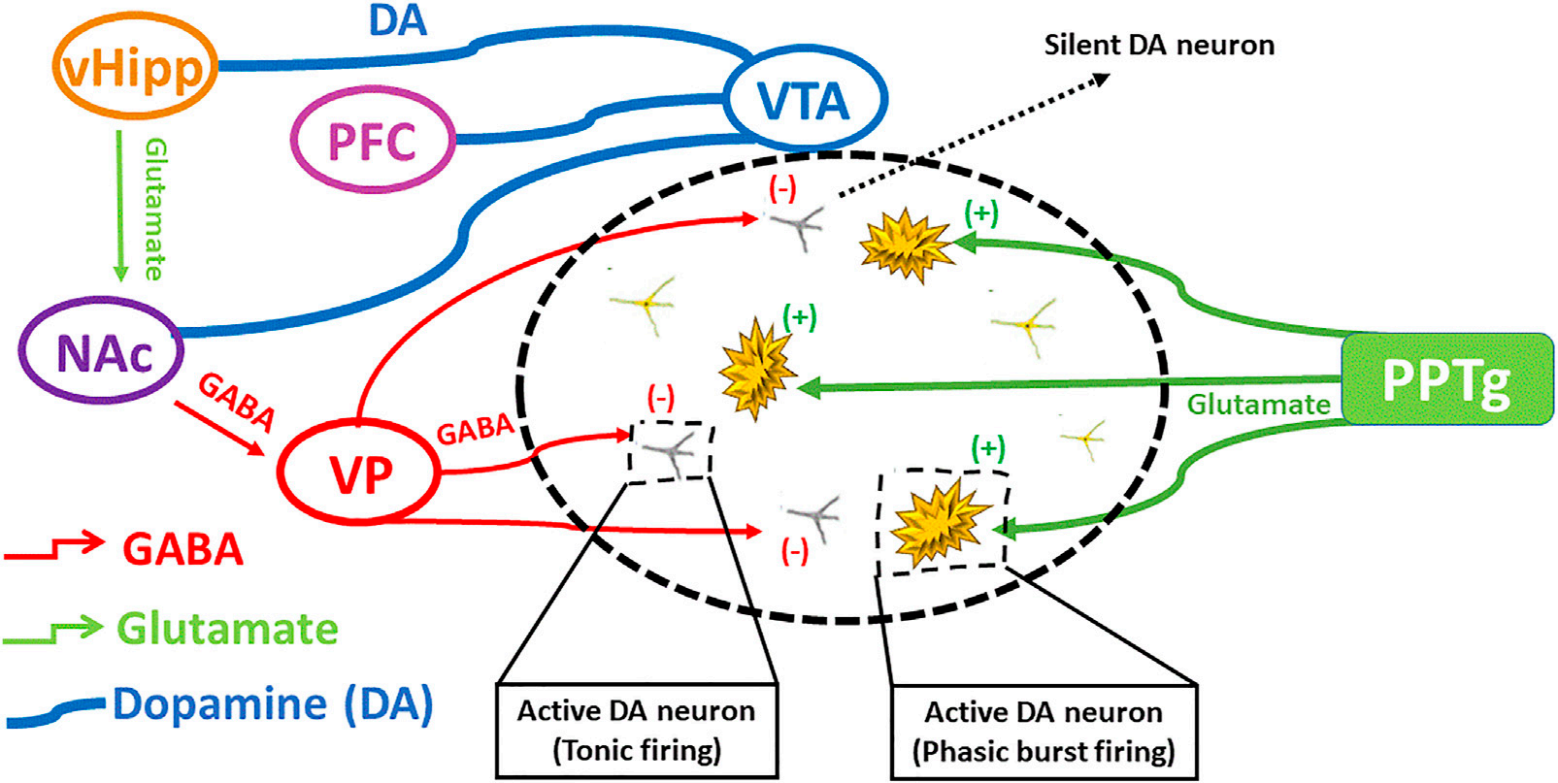
The interplay of different neuronal signals involved in schizophrenia. Ventral hippocampus regulates dopamine levels by excitatory projections towards ventral pallidum, that regulates GABAergic transmission producing silent DA neurons, influencing on DA rapid burst firing and tonic firing. Modified from (Grace and Gomes 2019).

Etiopathogenicity of schizophrenia is mainly understood based on several hypotheses (Fendri et al., 2006). The dopamine hypothesis remains a mainstay in understanding schizophrenia and is based on the fact that antipsychotics produce their effect by blocking dopamine D2/D3 receptors. It was further validated by the action of those agents which enhance dopamine level (Abi-Dargham, 2004). Hypo-stimulation of D1 receptor in hippocampus causes negative and cognitive symptoms, while hyper-stimulation of dopamine D2 receptor causes positive symptoms in the subcortical regions (Davis et al., 1991). However, new approaches during recent times have demonstrated a complex interplay among different neurotransmitter circuits (Agren et al., 1991). Another hypothesis indicated that the reduced function of NMDA receptors could produce symptoms of schizophrenia (Moghaddam, 2003; Coyle, 2006). A controlled study showed that antipsychotic drugs positively affected negative symptoms and to lesser extent, cognitive and positive symptoms by increasing the function of NMDA receptors (Coyle, 2006). Moreover, the role of glutamate in schizophrenia was depicted by the discovery of phencyclidine (PCP angel dust) as it induces a psychotic condition by powerful antagonistic action on glutamate receptor i.e. NMDA receptor (Lodge, 1989). The action of dopaminergic neurons may either directly be enhanced by the glutamatergic neurons or indirectly inhibited through the involvement of GABAergic transmission. The interplay of different neuronal signals involved in schizophrenia is depicted in Figure 2.

Dopamine, GABA and glutamate are not the only neurotransmitters involved, but serotonin and noradrenaline also play a significant role in the onset of disease. Moreover, many atypical antipsychotics possess adrenergic blocking ability (Hayes and Schulz, 1983). Lysergic acid diethylamide acts as a serotonin agonist on limbic cortex affecting GABAergic neurons that causes a reduction in glutamatergic tone in corticostriatal area resulting in hallucinations (Gellman and Aghajanian, 1991). Newer atypical antipsychotics have better tolerability profile as compared to typical antipsychotics owing to higher affinity for 5HT2A and lower affinity for D2 receptors in comparison to typical antipsychotic drugs (Schotte et al., 1996). Other receptors usually involved are 5HT2C, 5HT6 or 5HT7 and their modulation shows fewer extra pyramidal symptoms (EPS) (Meltzer and Fatemi, 1996). In recent studies, it was found that atypical anti-psychotic action was partly mediated through their agonistic action at 5HT1A and 5HT2C, and antagonistic action at 5HT6 and 5HT7 (Meltzer, 1999). Some antipsychotics such as phenothiazines, induce less EPS, which shows their effects were partially muscarinic antagonistic in nature. In striatum, dopaminergic terminals have an affinity to affect cholinergic interneurons that eventually affect D2 inhibitory receptors (Pisani et al., 2007). When an antipsychotic agent blocks D2 receptors, it enhances acetylcholine release in striatum. Indeed, it is also now considered that 5HT has no direct involvement in pathophysiology of schizophrenia, but its manipulation with D2 antagonism can produce improved therapeutic effects (Meltzer and Nash, 1991).

### Neurotransmitters and Brain Regions Involved in Schizophrenia

In addition to several neurotransmitters, there is also huge brain areas implicated in schizophrenia, including brainstem, striatum, limbic cortex, neocortex and basal ganglia (Grace and Gomes, 2019). Imaging studies have revealed the lateral and third ventricle enlargement, loss of some brain volume and, volume deficit in the prefrontal and temporal cortex, para-hippocampus, hippocampus and thalamus (Wright et al., 2000; Sullivan et al., 2003). Other cerebral lesions include cavum septi pellucid enlargement, and abnormalities in corpus collosum, cerebellar and basal ganglia (Niznikiewicz et al., 2003; Antonova et al., 2004; Honea et al., 2005). Moreover, cyto-architectural abnormalities in the grey matter of entorhinal area, corticolimbic portion, and aberrant neurons in the white matter of prefrontal cortex, para-hippocampus and temporal regions are evident (Arnold et al., 1997; Arnold et al., 2005).

**FIGURE 3:**
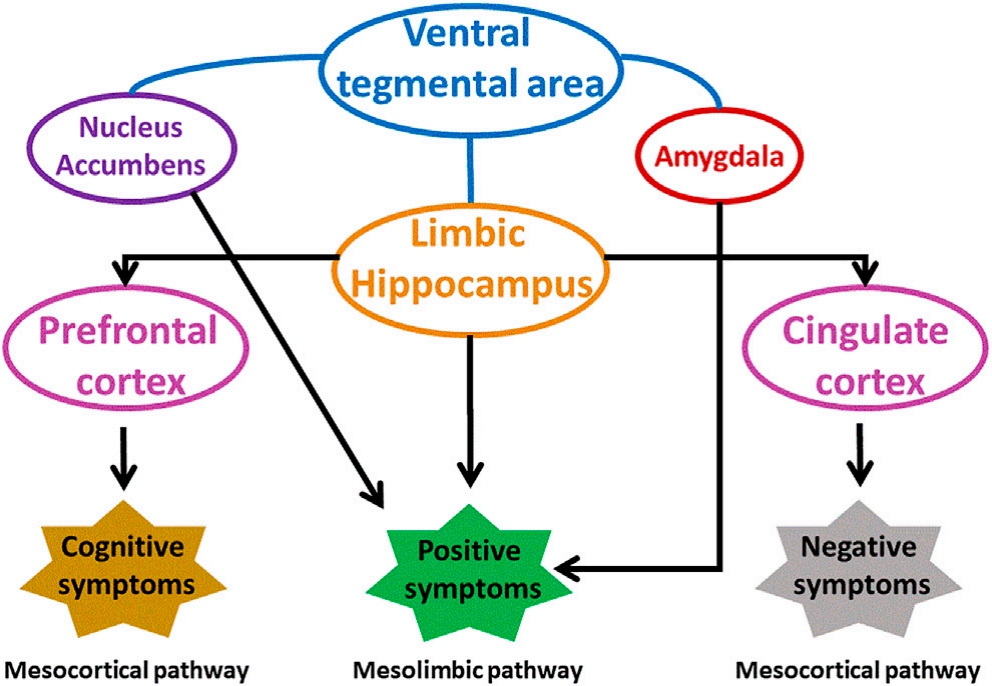
Involvement of different brain areas in pathophysiology of schizophrenia. Modified from (Grace and Gomes 2019). VP, ventral pallidum.

The abnormal dopamine signaling in striatum is responsible for increase of positive and negative symptoms, and decline of cognition in schizophrenia. Striatum mainly associative striatum acts as an integrative hub that moderates communication between limbic and motor regions. In schizophrenia, anomalous dopamine signaling in associative striatum adversely affects integrative functions, connectivity between striatum and cortex disrupting the cortical input from emotional, cognition and motor regions. Dopamine receptors respond differently to dopamine in different regions of striatum. An increased level of D2 receptors was found in associative striatum of schizophrenic patients that was responsible for cognitive deficit and altered neuronal information arriving from various areas of prefrontal cortex (McCutcheon et al., 2019; Simpson et al., 2010). Summary of various brain regions involved in schizophrenia is shown in Figure 3.

### Oxidative Stress, Inflammation and Immune System Involved in the Schizophrenia

Recent investigations showed that the oxidative stress and neuro-inflammation played a critical role in the pathogenesis of schizophrenia. Inflammation, oxidative stress and altered expression of proteins collectively lead to schizophrenia. Mitochondrial damage in neurons results in the impairment of mitochondrial respiration and changes in morphology. It also causes a low pH in the brain leading to psychotic symptoms and cognitive defects (Manji et al., 2012; Morris and Maes, 2014). These effects in peripheral tissues work as biomarker of the disease. Oxidative stress is evident both in early onset and chronic schizophrenia (Boskovic et al., 2011). The immune system acts by producing ROS and RNS promoting the release of cytokines that causes neuro-inflammation (Halliwell, 2012). By manipulation of these oxidative stress responses, different new pharmacological treatments can be identified.

An increased amount of ROS and RNS saturates ability of antioxidants, such as glutathione, which neutralize them to cause oxidative stress (Sies, 2015). Inflamosomes, after formation, can stimulate the production of IL-18 and IL-1B that adversely affect the microglia, macrophages and astrocytes. Interleukins also interact with the cytokines (Calabrese, 2008). Moreover, there is variable response to anti-schizophrenic treatments in patients. An increase in IL-6 is associated with delayed response as resistance to treatment is associated with an elevated level of both IL-6 receptor and Tumor necrosis factor receptor (TNFR) (Lurie, 2018). Furthermore, stress can increase pro-inflammatory cytokines leading to schizophrenia (Müller and Bechter, 2013). There is evidence that schizophrenic patients have increased level of peripheral plasma cytokines, prostaglandin E2, IL-1, IL-8, and C reactive protein, indicating elevated immune response in peripheral plasma (Miller et al., 2011). It is now established that the immune changes in peripheral blood are indicative of brain function and behavior in different neuropsychiatric disorders (Dantzer, 2004). Understanding the causes and mechanism of neuro-inflammation associated with schizophrenia presents a potential target for the treatment of disease (Watkins and Andrews, 2016).

The involvement of abnormal immune response in pathogenesis of disease is evident (Feigenson et al., 2014). Traditionally, it is thought that the brain is protected immunologically by blood brain barrier, but recent studies demonstrated a complex interaction between brain, systemic inflammation and immune system, altering the mood and behavior (Khandaker et al., 2015). Moreover, alterations in immune system can profoundly affect neurotransmission involved in the pathogenesis of schizophrenia. It can activate the enzyme indoleamine 2,3-dioxygenase involved in tryptophan and kynurenic acid metabolism that influences glutamatergic and serotonergic neurotransmission through these neuroactive metabolites (Müller and Bechter, 2013).

### Cholinergic System as Potential Target

Cholinergic system is a potential target for ameliorating the symptoms of schizophrenia, including negative and cognitive symptoms. This system affects working memory, attention and motivation (Berman et al., 2007). As, Nicotinic acetylcholine receptors (nAChR) belong to family of ligand gated ion channel and its homomeric subtype, nAChR-α7 is found in central and peripheral nervous system. It found to have pivotal role in the pathophysiology of several neurological disorders including psychosis. There is a direct role of nAChR-α7 and muscarinic M1 receptor in schizophrenia symptoms (Ochoa and Lasalde-Dominicci 2007; Raedler et al., 2007). Hence, agents acting on these targets are potential candidate for treating schizophrenia.

### Phosphodiesterase Inhibitors for Schizophrenia

Phosphodiesterases (PDE) are the enzymes often targeted for their pharmacological inhibition because PDE inhibitors can potentiate the effect of different physiological processes which are mediated by cGMP or cAMP. These are identified as a new adjunctive therapy for different diseases including schizophrenia (Madeswaran et al., 2012).

### Signaling Pathways as Treatment Target for Schizophrenia

It is found that the level of glycogen synthase kinase-3 (GSK-3) is increased while Akt (protein kinase) is reduced in schizophrenic patients (Sahin et al., 2014). Dopamine regulates lithium sensitive signaling cascade that involves GSK3β. Modulation in its increased activity can have impact on long lasting remission of the disease (Duda et al., 2018). A protein kinase, called Akt, is involved in a variety of functions such as neuronal cell size regulation, synaptic plasticity and cell survival, while Akt1 has the most important role in schizophrenia. It is activated by phosphorylation. The GSK-3, regulating the synaptic plasticity, is inactivated after phosphorylating with Akt1. The reduced level of Akt1 also decreases phosphorylation of GSK, thus the activity of GSK-isoform GSK-3 β is enhanced in the frontal cortex area of schizophrenic patients. Antipsychotic drugs are expected to increase Akt activity through blockade of D2 receptor activation (Karam et al., 2010). Other molecules such as Wnt, are lipoglycoproteins, which regulate embryonic development by acting as signaling molecules. Dysregulation in the Wnt signaling pathway contributes to various human diseases including schizophrenia. Moreover, reduction of β-catenin, increase in Wnt-1 expression and reduced GSK-3β contribute to multifaceted kinase present in Wnt signaling (Hoseth et al., 2018). Investigations are being carried out on natural and synthetic agents affecting these cascades in order to cope with schizophrenia.

### Alteration in Hormonal Balance for Treatment Option of Schizophrenia

The protective role of estrogen against schizophrenia has been demonstrated previously. Recent clinical trials have validated the usefulness of estradiol in the treatment of disease (Gogos et al., 2015). Oxytocin and vasopressin have been implicated in the disease etiology and the antagonistic approach against vasopressin V1A receptors may provide an opportunity for treating schizophrenia (Park et al., 2020).

### Current Pharmacological and Non-pharmacological Treatments

Antipsychotic drugs are also called neuroleptics (meaning, taking hold of one’s nerves) and are used to manage acute and chronic schizophrenia symptoms. Generally, first generation (typical) and second generation (atypical) anti-psychotic drugs are used in allopathy (Khandakeret al., 2015). Typical antipsychotics include chlorpromazine, thioridazine, fluphenazine, trifluperidol, flupenthixol, loxapine, triflupromazine, trifluperazine, haloperidol, penfluridol and pimozide. Risperidone, clozapine, aripiprazole, olanzapine, ziprasidone, quetiapine and sulpiride are a few examples of atypical antipsychotic drugs. Atypical antipsychotics are generally more effective, but have fewer side effects as compared to typical anti-psychotic drugs [27]. General adverse effects of these synthetic drugs include but not limited to diabetes, dizziness, tardive dyskinesia, weight gain, sexual dysfunction, neuroleptic malignant syndrome, sedation and agitation. For avoiding these drug related problems, more efficacious and safer remedies are needed [36].

Several non-pharmacological treatments are currently used for the management of schizophrenia. Aromatherapy and aroma massage are helpful in ameliorating depressive symptoms of schizophrenia [54]. Acupuncture activates different brain regions involved in controlling emotions of schizophrenic patients [42]. For improvement of constant negative symptoms, loving kindness mediation (LKM) has been proved useful [39]. Yoga and aerobic exercises are also very helpful for the psychiatric symptoms of schizophrenia [49].

### Phytochemicals and Their Role in Schizophrenia

About 80% of the total population of Asia and Africa are dependent on natural therapeutics. Mainstream antipsychotic drugs are associated with several adverse effects. Therefore, several phytochemicals have been investigated for neuroprotection and anti-psychotic action in cell culture and animal models of CNS disorders. The vast majority of studies have demonstrated that the anti-psychotic and neuroprotective action of phytochemicals is due to their antioxidant action [19]. As pathophysiology of schizophrenia clearly depicts oxidative burden in brain, so the natural antioxidants in the form of extracts or individual phytochemicals are effective for treatment of schizophrenia. These phytochemicals have gained attention due to their therapeutic value, less adverse effects, better safety profile and high efficacy (Gao and Snyder 2013; Choudhury et al., 2014). Several phytochemicals investigated in pre-clinical and clinical studies are shown in Table 1 and 2.

**TABLE 1:** Antipsychotic potential of phytochemicals in non-clinical studies.

Chemical Class	Phytochemical	Source	Assay/Test	Animal/ Cell Type	Doses	Method	Mechanism	Result	References
Alkaloids	Arecoline	*Areca catechu*	Y-maze Behavioral test	cuprizone induced mouse model	0, 2.5, or 5 mg/kg/Day	Recorded spontaneous alternation behavior	Preventing white matter injury, prevented memory impairment	Attenuated spatial working memory impairment, increased the expression of myelin basic protein in the frontal cortex	Xu, Adilijiang, Wang, You, Lin, Li and He (2019)
	Stepholidine	*Stephania intermedia*	Paw test Pre-pulse inhibition	Male Wistar rats	4–16 mg/kg	Determined limb retraction time and	D1 receptor agonist and D2 receptor antagonist	Increased hind limb retraction time Reverse apomorphine induced disruption	Ellenbroek, ZHANG and JIN (2006)
	Galantamine	*Galanthus caucasicus*	Dopamine receptor agonism by apomorphine, NMDA antagonism by MK-801, muscarinic receptor antagonism by scopolamine	Wistar rats	0.3, 1.0 and 3.0 mg/kg	Apomorphine agonism, NMDA and Ach antagonism models	Increase in cholinergic activity	Pre-pulse inhibition was improved	Hohnadel et al. (2007)
	Corymine	*Hunter zeylancia*	2 electrode voltage clamp technique	cDNA clones of NR1a and NR2b OF Xenopus	100 µM	Potentiating effect of corymine was induced in presence of glycine	Potentiation on NMDA response	Potentiates the NMDA induced currents and can be used for schizophrenia	Leewanich et al. (2005)
	Reticuline	*Ocotea duckei*	Amphetamine induced hyper-motility	Swiss albino mice	50–100 mg/kg	Number of steps recorded	Dopamine antagonist activity	Reduced hyper-motility	Morais, Barbosa-Filho and Almeida (1998)
	Geissoschizine methyl ether	*Uncariae ramulus*	Cell based Calcium imaging analysis	Human cell line and mouse brain tissue		Inhibited dopamine induced calcium response	Partial agonist/antagonist at D2, partial antagonist at 5HT receptors	Inhibited calcium induced serotonin current	Ueda et al. (2011)
	Psychollatine	*Psychotria umbellata*	Male adult mice	a) Apomorphine induced climbing b)MK-801 induced hyperactivity	100 mg/kg	Climbing behavior and locomotion determined	Interference with DA, 5HT and NMDA receptors	Attenuated the climbing and locomotion	Costa-Campos, Lara, Nunes and Elisabetsky (1998)
	Alstonine	*Picralima nitida*	Male albino mice Male Wistar rats	a)Apomorphine induced stereotypy, b)haloperidol induced catatonia	0.5 to 2 mg/kg	Determined behavioral score and catatonic time	Modulating the DA uptake and serotonin receptors	Reduction in behavioral score, diminished catatonic time	Costa-Campos, Lara, Nunes and Elisabetsky (1998)
	Physostigmine	*Physostigma venenosum*	Conditioned emotional response	Male Wistar rats	0.5 mg/kg	Pre-exposure and in conditioning response is measured	Induced Latent inhibition disruption	Reverse the cognitive impairment in schizophrenia	Barak and Weiner (2010)
Amino acid and derivatives	Leucine	*Cucurbeta pepo*	Apomorphine induced stereotypy. Haloperidol induced catalepsy	Wistar rats	0.7 mg/kg	Stereotypy, catalepsy	Anti-dopaminergic effect	Decreased stereotypy, potentiated catalepsy	Suresh and Raju (2013)
	Betaine	*Beta vulgaris*	PPI, NOR	Male ICR mice	O, 30, 100 mg/kg	%PPI, recognition index %	Modulation of NMDA R glycine site	Attenuated ketamine induced disruption in PPI, improved novel recognition	Lin et al. (2016)
Bioflavonoids/ Polyphenols	Quercetin-3- rutoside	*Fagopyrum esculentum*	PCR-RFLP method	Human brain	10 µmol	Hetero-plasmic sequence variation determined	reduced oxidative stress	Quench the superoxide production	Marchbanks et al. (2003)
	Scopoletin	*Morinda citrifolia*	a) Apomorphine induced Cage climbing, b) Amphetamine induced stereotypy	Male ICR mice	0.1 mg/kg	Climbing and stereotypy determined	Anti-dopaminergic effect	Reduction in climbing and stereotypy	Pandy and VijeePallam (2017)
	Quercitin	*Lonchocarpus cyanescens*	Novel object recognition (NOR)	Balb-C mice	25 and 50 mg/kg	Memory impairment model by ketamine used	Antioxidant potential	Improves cognitive deficit	Mert, Turgut, Arslanbas, Gungor and Kara (2019)
	Myricitrin	*Eugenia uniflora*	Apomorphine induced stereotypy, catalepsy and paw test	Swiss albino mice Wistar rats	5,10 and 30 mg/kg	Stereotypy, climbing, limbs retraction and catalepsy noted	Nitric oxide and Protein kinase C inhibitor	Blocked stereotypy, climbing, impaired retraction time of limbs, increased catalepsy	Moore and Martin (1957); Pereira et al. (2011)
Cannabinoids	Cannabidiol	*Cannabis sativa L*	Apomorphine induced stereotypy	Male Wistar rats	15–480 mg/kg	Stereotypy and prolactin secretion were measured	Blockade of serotonin reuptake or increased GABAergic activity	Attenuated the stereotypy and increase in prolactin	Zuardi et al. (1991)
Carotenoids	Crocin	*Crocus sativus L*	MK-801 induced Rotarod test, open field test	Neonatal Sprague Dawley rats	25,50 mg/kg	Balance, motor coordination and locomotion	regulations of SIRT1 and downstream BDNF expression in the hippocampus	Improved motor coordination, balance and locomotion deficit	Sun, Zhao, Xie and Wan (2020)
Cholesterols	Hydroxytyrosol	*Olea europaea*	Prenatal restraint stress model	Sprague dawley rats	10 and 50 mg/kg/day	Spontaneous alteration performance, Morris water maze test performed	Antioxidant, anti-inflammatory and brain protecting	Improves cognitive functions and Might be used for schizophrenia	Young et al. (2008); Calabrese et al. (2017)
	hydroxytyrosol	*Olea europaea*	Determination of DNA strand breakage (comet assay)	IMR-32 cell line; histiocytic lymphoma U937 cell line		extent of H2O2-induced DNA damage	Decrease DNA damage	Neuroprotective efficacy, might be useful for schizophrenia	
Flavonoids/ Polyphenols	Naringin	*Citrus paradisi*	Locomotor activity, PPI	Male Wistar rats	100 mg/kg	Counts per 5 min, %PPI	targeting Wnt/β-catenin together with Akt/GSK-3β pathways	Increased locomotor activity, increased %PPI	George et al. (2020)
	Nobiletin	*Citrus depressa*	MK-801 induced learning impairment	ddY mice	2–50 mg/kg	Step-through Passive-Avoidance Task	Improves hypo-function of NMDA receptor-ERK signaling	Improvement of cognitive symptoms, might be beneficial for schizophrenia	Naka et al. (2007)
Glycosides	Bacosides A and B	*Bacopa monnieri*	Novel object recognition test	Rat		Discrimination ratio was obtained	Increasing VGLUT2 density to normal level	Increase in Discrimination Ratio score	Wetchateng and Piyabhan (2014)
	sulforaphane	*Brassica oleracea*	Locomotor activity, Pre-pulse inhibition	Male mice	30 mg/kg	Hyper-locomotion and PPI deficits were examined	An antioxidant protects against dopaminergic neurotoxicity by increasing Nrf2 expression	Attenuated PCP-induced hyper locomotion and PPI deficits	Shirai et al. (2012)
	Hypericin	*Hypericum perfolatum*					Inhibit D3/D4	Might be used for schizophrenia	Butterweck et al. (2002)
	Emodin	*Rheum rhabarbarum*	Acoustic startle response, Methamphetamine induced hyper-locomotion	Sprague dawley rats	50 mg/kg	Startle response and locomotor activity	Targets ErbB signaling alters dopamine and serotonin metabolism	Suppressed acoustic startle response and hyper-locomotion	Mizuno et al. (2008); Mizuno et al. (2010)
	cardenolides	*Nerium oleander*		NMRI male albino mice				Might be used for schizophrenia	Zhang (2004)
	Polygalasaponins	*Polygala tenuifolia*		a) Female TO mice	25–500mg/kg	Animal’s movements, behavioral patterns and hyperactivity measured	Dopamine and serotonin antagonist activity	Reduction in climbing, stereotypy and hyperactivity	Chung et al. (2002)
b) Male lister hooded rats									
	Diosmin	*Scrophularia nodosa*	Apomorphine induced stereotypy, catalepsy	Swiss male mice	25, 50, 100 mg/kg	Stereotypy scoring, cataleptic behavior	Enhancement of GABAergic neurotransmission	Attenuated stereotypy, devoid of cat	Eneni et al. (2020)
	Picroside II, wedelolactone, 7-o-methylwogonin and isoformononetin	*Picrorhiza scrophulariiflora*		*In vitro* studies		Docking studies	Interaction with NMDA receptor	Good docking score	Bagchi and Somashekhar (2014)
Polyphenols	Resveratrol	*Vitis vinifera*	a) Apomorphine induced stereotypy	Swiss albino mice	200 and 400 mg/kg	Stereotypy and grooming determined	D1 receptor antagonistic effect	Decreased climbing and swim induced grooming	Magaji et al. (2017)
	b) Swim induced grooming								
	Kaempferol	*Lonchocarpus cyanescens*	a) Amphetamine induced-stereotype	Wistar rats albino mice	50–400 mg/kg (i.p)	Stereotypy was measured, Spontaneous motor activity was measured		Suppressed stereotyped behavior. Reduction in spontaneous motor activity	Sonibare et al. (2012)
	b) Open field test								
	Rutin	*Morinda citrifolia*	a) Apomorphine induced Cage climbing	Male ICR mice	0.1 mg/kg	Climbing and stereotypy determined	Inhibition of D2 receptors	Reduction in climbing and stereotypy	Pandy and VijeePallam, (2017)
			b) Amphetamine induced stereotypy						
	Curcumin	*Curcuma lona*	Assay based on tietze method	Mice	10 and 50 µM	Oxidized and reduced GSH level determined	Antioxidant action	Increased GSL and GSH level in astrocytes and neurons	Lavoie et al. (2009)
	Genistein	*Genista tinctoria L*	Locomotor activity, forced swim test, active avoidance	rats	50 mg/kg	Hyperactivity, immobility, avoidance	Anti-dopaminergic activity due to increased estrogen	Hyperactivity, enhanced immobility and decreased avoidance	Kalpana, Raju and Merugu (2014)
	Gallic acid	*Camellia sinensis*	ketamine-induced psychosis	Swiss albino mice	50, 100 and 200 mg/kg	Stereotypy, locomotor activity	enhancement of NMDA receptor function	Stereotypy improved and locomotor activity increased	Yadav et al. (2018a)
	Morin	*Allium cepa*	Open field, apomorphine-induced stereotypy, ketamine-induced stereotypy	Male Swiss mice	50 and 100 mg/kg	Locomotor activity, stereotypy	Might be enhancement of GABA activity	reduced spontaneous locomotor activity. Also, morin suppressed apomorphine-induced stereotypy and ketamine induced stereotypy	Ben-Azu et al. (2018)
Polypropanoid	Alpha (α)—asarone	*Acorus calamus*	Apomorphine-induced stereotypy	Swiss albino mice	30 and 50 mg/kg	Climbing time and climbing behavior determined	Anti-dopaminergic property	Decrease in the cage climbing time and climbing behavior	Pandy and Vijeepallam (2016)
Sesquiterpene	Tutin	*Coriaria ruscifolia*	Ca2+ transients& CREB analysis	Mouse spinal cord neurons	1, 3, 5 and 8 mg/kg		Inhibit GABA A receptor	Might be used or schizophrenia	Fuentealba et al. (2007)
Steroids	Anaferine, Beta-Sitosterol, Withaferin A, Withanolide A, Withanolide B and Withanolide D	*Withania somnifera*		Molecular docking			Inhibition of GluN2B-containing NMDARs	Might be useful for schizophrenia	Kumar and Patnaik (2016)
Sterol	Stigmasterol	*Akebia quinata*	Ketamine induced stereotypy	Swiss albino mice	50mg/kg	Stereotypy and hyperlocomotion measured	Antioxidant action and increase in GABA and decrease in dopamine and acetylcholine	Decrease in stereotypy and locomotion	Yadav et al. (2018b)
Terpenoid	1,8-cineole	*Hyptis martiusii*	haloperidol-induced catalepsy, and ketamine-induced hyperkinesia	female Swiss mice	50 mg/kg	Catalepsy and hyperkinesis	Possible modulation of dopaminergic and glutamatergic systems	potentiated haloperidol-induced catalepsy and reduced ketamine-induced hyperkinesia	Monteiro et al. (2019)
Xanthone/ Polyphenol	α-mangostin	*Garcinia mangostana* L	Pre-pulse inhibition (PPI) test, open field test (OFT), forced swim test (FST)	Sprague Dawley dams and offsprings	20 mg/kg	Sensorimotor gating, locomotor activity and depressive behavior determine	antioxidant	%PPI, locomotor hyperactivity and depressive like behavior were reversed	Lotter (2018)
	Magniferin	*Magnifera indica*	Open field test	Swiss mice, Wistar rat	50 mg/k g	Locomotor behavioral changes	Antioxidant, anti-inflammatory effect	Overcome grooming and stereotypy	Rao et al. (2012)

**TABLE 2:** Antipsychotic potential of phytochemicals in clinical studies.

Chemical Class	Phytochemical	Source	Assessments	Dose	Study Design	Mechanism	Result	References
Alkaloids	Apomorphine	*Nymphea caerulea*	Interview using NHSI (new haven schizophrenia index)	1.5 to 6 mg	Randomized double blind placebo study	Potent effect on presynaptic dopamine receptors in addition to its postsynaptic stimulation	Decrease in psychotic symptoms in chronic patients	Smith et al. (1977); Fletcher, Frith et al. (1996)
	Reserpine	*Rauolfia serpentina*	Seven-point behavioral rating scale	1–8 mg/day	Controlled study of 8 months	Depressor of hypothalamus and facilitator of synaptic transmission	Marked improvement in behavior occurred	Cowden et al. (1955)
	Nicotine	*Nicotiana tabacum*	Profile of mood states (POMS) and continuous performance test (CPT)	7 mg/day	N/A	Alpha 7 nicotinic receptor agonist	Attentional function is increased	(Levin, Conners, Silva, Hinton, Meck, March and Rose 1998)
Amino acid and derivatives	Glycine	*Glycine max*	PANSS and Scale for assessing Negative Symptoms (SANS)	0.14 to 0.8 g/kg/day	Open label trial	Potentiate NMDA transmission	Improvement in negative symptoms	Leiderman et al. (1996)
	Sarcosine	*Arachis hypogaea*	Positive and Negative Syndrome Scale total score	1–2 g/day for six weeks add on therapy	Double blind randomized clinical trial	Glycine 1 transport inhibitor, increases N-methyl-d-aspartate transmission	Reduced positive and negative syndromes in anti-psychotic naïve patients	Lane et al. (2008)
Cannabinoids	Tetrahydrocannabinol	*Cannabis ruderalis*	Clinical global impression, brief psychiatric rating scale	2.5 to 10 mg twice a day	Clinical case study	Affecting endocannabinoid receptors	Refractory schizophrenia symptoms improved	Schwarcz et al. (2009)
	Cannabidiol	*Cannabis sativa*	Positive and negative symptoms, Global assessment of functioning scale	1000 mg/day for six weeks	Double blind randomized clinical trial	As adjunct therapy	Reduced positive symptoms and improved cognitive performance	Philip McGuirePsych. et al. (2018)
Flavonoids/ Polyphenols	Luteolin	*Salvia rosmarinus*	N/A	N/A	N/A	Modifier of NMDA function	Schizophrenic symptoms decreased	Hannan et al. (2021)
	Apigenin	*Perilla fruitiscenscens*	N/A	N/A	N/A	Restore function of NMDA receptor by modulating hSKCa3 channel	Schizophrenic symptoms decreased	Hannan et al. (2021)
Phenolic acid and derivatives	Sodium benzoate	*Styrax benzoin*	Assessment of positive and negative symptoms, and clinical global impression in treatment refractory schizophrenia	0.5 g twice a day for 12 weeks	Double blind randomized clinical trial	Adjunctive use	Lack of efficacy in patients with early psychosis	Scott et al. (2020)
	Sodium benzoate	*Styrax benzoin*	Clinical Global Impression (CGI), assessment of negative symptoms	1 g/day for six weeks add-on therapy	Double blind randomized clinical trial	d-amino acid oxidase inhibitor	Improvement of clinical symptoms and recognition	Lane et al. (2013)
Polyphenols	Resveratrol	*Vitis vinifera*	positive and negative symptoms scale and extrapyramidal symptoms scale	200 mg/day for eight weeks add on therapy	Double blind randomized clinical trial		Managed negative symptoms and increased efficacy of risperidone	Samaei et al. (2020)
Sesquiterpenoids	Caryophyllene	*Cannabis sativa*	N/A	25 to about 100 mg add-on therapy	Clinical trials (application N° EP13763464.8A)	CB2-selective phyto-cannabinoid	Might improve schizophrenia symptoms	Anavi-goffer and Gertsch (2015)

**FIGURE 4:**
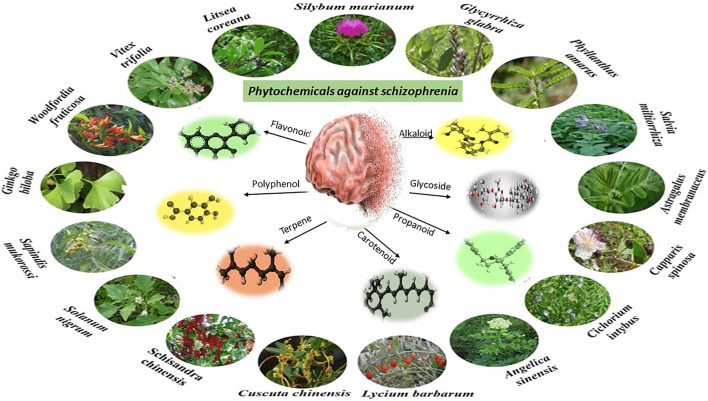
Phytochemical classes effective against Schizophrenia.

Phytochemicals showing efficacy against schizophrenia belong to different phytochemical classes such as alkaloids, tannins, glycosides, phenolic acids, flavonoids, terpenes, terpenoids and essential oils. Theses phytochemical classes are summarized in Figure 4.

### Alkaloids

Alkaloids are present in all plant parts, especially in flowers (Girdhar et al., 2015). These are mainly useful in treating several neurodegenerative disorders. These phytochemicals are effective against schizophrenia via affecting acetylcholine concentration, increasing GABA, antagonizing NMDA receptors, anti-oxidant action, anti-amyloid activity and, preventing neuro-inflammation (Dey and Mukherjee, 2018). Several alkaloids have now been investigated for treatment of schizophrenia. Arecoline, a pyridine alkaloid, has shown a capacity for muscarinic receptors as cholinergic agonist and can improve cognitive symptoms in schizophrenic patients. It also exerts antioxidant action and prevents demyelination of the cerebral white matter to attenuate memory impairment (Xu et al., 2019). Stepholidine, a protoberberine alkaloid, has a special feature of combined D1 agonist and D2 antagonist effect, and is useful in improving memory deficit in schizophrenia (Ellenbroek et al., 2006). Aporphine alkaloids, including apomorphine, reportedly cause amelioration of schizophrenic symptoms in patients by potently antagonizing dopamine at its receptor site (Smith et al., 1977; Fletcher et al., 1996). Isoquinoline alkaloids have also been investigated against schizophrenia. Galantamine increases the NMDA current in the rat cortical neurons. It also enhanced the effects of Ach by positive modulation of nAchR that decreased the attentional impairment and, increased short term memory and attention (Moriguchi et al., 2005; Schubert et al., 2006). A combination of galantamine and memantine was effective to enhance cognition in schizophrenic patients (Koola et al., 2014). Reticuline has also demonstrated antipsychotic activity through anti-dopaminergic actions (Morais et al., 1998).

Nicotine, a pyridine alkaloid, was effective in schizophrenic patients to improve attention deficit through action as an alpha nicotinic receptor agonist (Levin et al., 1998). Some indole alkaloids, such as corymine, potentiated the NMDA current and showed efficacy against schizophrenia, while alstonine showed anti-schizophrenic effect by modulating the dopamine uptake and NMDA receptor. These alkaloids also reduced behavioral problems of schizophrenic patients (Costa-Campos et al., 1998). The ameliorating effect of geissoschinzine methyl ether against schizophrenia also occured through modulation of dopamine receptors as well as partial antagonistic effect against NMDA receptors.

### Glycosides

In glycosides, a sugar moity is attached to non sugar molecule through glycosidic linkage. Glycosides are present in plants as secondary metabolites and are their “offense and defence” components (Shadkami and Jones, 2012; Wang et al., 2016). A study on use of bacoside A and B isolated from *Bacopa monnieri* has shown an improvement in cognitive defects in schizophrenic model by increasing vesicular glutamate transporter 2 in cingulate gyrus region (Wetchateng and Piyabhan, 2014). Isothiocyanates, such as sulforaphane, exhibited antipsychotic activity through activation of Nrf2 pathway, detoxification of phase 2 enzymes and, antioxidant action by enhancing electrophilic response elements (Shirai et al., 2012). Hypericin is nephthodianthrone and exhibits antioxidant properties. It inhibits D3/D4 receptors and is a candidate drug for the management of schizophrenia (Butterweck et al., 2002; Saranya et al., 2019). Emodin Targets ErbB signaling and alters dopamine and serotonin metabolism to exhibit ameliorating effects against schizophrenia symptoms (Mizuno et al., 2008; Mizuno et al., 2010). Polygalasaponin, a saponin glycoside, has anti-schizophrenic activity due to its dopamine and serotonin antagonist activities (Chung et al., 2002). It is also found that iridoid glycosides and cardenolides were effective in treating psychotic symptoms that required further investigation. Beta sitosterol also inhibited the GluN2B-containing NMDA receptors as demonstrated by docking studies. Picroside II also showed *in vitro* potential of antipsychotic activity (Bagchi and Somashekhar 2014; Kumar and Patnaik 2016).

### Polyphenols

Polyphenols are plant secondary metabolites that have demonstrated neuroprotective and anti-schizophrenic activity. Several studies have indicated that these are useful against neurologic and psychotic disorders (Wu et al., 2009). Kaempferol has demonstrated neuroprotective effects against schizophrenia due to its anti-infammatory, antioxidant and anti-apoptotic effects (El-Kott et al., 2020). Baicalin is reported to ameliorate negative symptoms and cognitive dyfunction in psychosis. This psychotic effect may be attributed to its anti-prolyl-oligopeptidase, anti-inflammatory and antioxidant actions (Miao et al., 2020; Tarragó et al., 2008).

Quercitin, a bioflavonoid, has potential to improve symptoms of schizophrenia due to its free radical scavenging activity (Mert et al., 2019). Myricitrin is an inhibitor of nitric oxide and Protein Kinase C. Its anti-schizophrenic effect is attributed to antioxidant action (Amanzadeh et al., 2019). Scopoletin and rutin are useful for alleviation of positive symptoms of schizophrenia due to the inhibitory interaction with D2 receptor (Pandy and VijeePallam 2017). Xanthones, such as α-mangostin and magniferin, have also been studied for anti-schizophrenic activity. α-mangostin is an antioxidant and has anti-inflammatory properties. It also inhibited phosphodiesterases and 5HT_2_A receptors, and was shown to be effective in rodent models of schizophrenia. Magniferin improved cognition by its antioxidant mechanism, preserving mitochondrial functions, anti-inflammatory activity and, reduction of dopamine (Lotter 2018; Lum et al., 2020), Hydroxytyrosol is a cholesterol which showed neuroprotection for multiple neurological and psychological diseases. It decreased oxidative stress by activation of Nrf2 pathway and enhanced the mitochondrial functions. It restored the learning ability and memory in prenatal stressed animal and human off springs, when administered during pregnancy, showing its vitality for preserving neurogenesis and cognitive functions in off springs (Chen and Wei, 2021).

Curcumin is known for its several beneficial effects on the nervous system attributed to its ability to raise the level of reduced glutathione (Lavoie et al., 2009; Miodownik et al., 2019). Curcumin exerted add-on effects of regular anti-psychotic drugs in chronic schizophrenic patients. Such treatments have shown improvement in negative symptoms of schizophrenia. Curcumin regulates the gene expressions involved in inflammation and modulates NMDA activity, which are associated with symptoms of schizophrenia. Morin also exhibited anti-psychotic like effects, without exerting extrapyramidal side effect, by enhancing GABA activity (Ben-Azu et al., 2018). Gallic acid also plays protective role against psychotic like behaviour through enhancement of NMDA receptor (Yadav et al., 2018b). Nobiletin, a flavonoid, improves hypo-functioning of NMDA receptors by acting on extracellular signal-regulated kinases (ERK) signaling and ameliorates cognitive symptoms of schizophrenia (Nakajima et al., 2007).

Diosmin, a flavone, enhances GABA transmission to treat symptoms of schizophrenia (Eneni et al., 2020). Naringin is a flavonoid that acts on Wnt/β catenin and Akt/GSK-3β pathways to exert anti-schizophrenic effect (George et al., 2020). It is also found that genistein, an isoflvone, had exhibited therapeutic effects against different symptoms of schizophrenia by acting on estrogen receptor and affecting dopamine pathway (Kalpana et al., 2014). Furthermore, both apigenin and luteolin have demonstrated considerable potential to improve the symptoms of schizophrenia (Zhao and So, 2018).

### Terpenes and Terpenoids

Tutin is a sesquiterpene that inhibited glycinergic activity and blocked GABA-A receptors. Moreover, 1,8 cineole is amonoterpenoid that acts on dopamine and glutamate pathways. Caryophylline is a sesquiterpene isolated from essential oils that acts as phytocannabinoid and is being effectively investigated in clinical research of schizophrenia (Kucerova et al., 2014).

### Cannabinoids

Cannabinoids belong to terpenoid class and are helful in the treatment of neurodegenerative diseases. Results of a meta-analysis have concluded that the patients of schizophrenia have increased amount of endocannabinoid anandamide in their blood, cerebrospinal fluids and cannabinoid 1 receptors (CB1) present on immune cells (Davies and Bhattacharyya, 2019). Three randomized trials have reported the reduction in disease positive symptom and improved cognition by using cannabidiol (White, 2019).

Cannabidiol is a cannabinoid that blocks the serotonin uptake and increases GABAergic activity to exert anti-schizophrenic effect. This effect was also evident in schizophrenic patients who used cannabis (Morgan and Curran, 2008). Moreover, cannabidiol had also shown a clear advantage in clinical studies over other antipsychotics as it did not exhibit any movement like problems associated with the use of other antipsychotics (García-Gutiérrez et al., 2020).

Another cannabinoid, tetrahydrocannabinol, also improved the symptoms of schizophrenia due to its effect on the endocannabinoid receptors (Schwarcz et al., 2009). On the other hand in some reports suggested that the 9-tetrahydrocannabinol administration had increased the symptoms of psychosis. But researchers reported that tetrahydrocanabinol might have dose dependent effect. As at low doses, it improved the symptoms of psychosis while it inflicted disruption to brain circuits causing worsening of psychotic symptoms at large doses.

### Phytosterols

Phytosterols and oxyphytosterols (oxidation products of phytosterol) are naturally synthesized by several plants. Exposure of these natural agents is growing due to increased intake of plant food enriched with phytosterol and oxyphytosterol (Jie et al., 2020).

Stigmasterol is a phytosterol present in vegetables, legumes, nuts, herbs and seeds. It is shown to inhibit ketamine induced biochemical, histopathological and behavioral alterations in mice to exhibit antipsychotic potential. It manages psychosis by ameliorating inflammation and oxidative stress, and by altering dopaminergic, acetylcholinergic and GABAergic neurotransmission (Yadav et al., 2018a).

### Carotenoids

Saffron (*Crocus sativus* L.) and its active constituents such as crocins and safranal have shown high potential for treatment of various central nervous system disorders such as anxiety, depression and memory defficit (Pitsikas, 2015). Crocin is a carotenoid that showed effectiveness as antipsychotic drug by regulating Brain-derived neurotrophic factor (BDNF) in hippocampus (Sun et al., 2020). There are increasing preclinical evidences that crocins reversed the ketamine induced memory deficit, hypermotility and social isolation at 15–50 mg/kg dosage in rats (Georgiadou et al., 2014). It is also found that crocins had inhibited the apomorphine induced deficit in novel object recognition task associated with dopaminergic dysfunction in rats (Pitsikas and Tarantilis, 2017). Based on better safety profile and the preclinical evidences of efficacy against psychosis, there is strong need for controlled clinical studies of these agents agianst schizophrenia (Pitsikas, 2021).

### Other Phytochemicals

Alpha asarone belongs to polypropanoid class of essential oils and exerts anti-schizophrenic activity due to antagonism of dopamine D2 and/or D1 receptors (Pandy and Vijeepallam, 2016). Glycine is an amino acid which improved the negative symptoms of schizophrenia in an open trial on human. This effect is attributed to its potentiating effect on NMDA receptors (Leiderman et al., 1996). It is found effective against treatment resistant schizophrenia, negative symptoms and cognitive problems when given as adjuvant to other medical therapies (Heresco-Levy et al., 2004). Leucine is also an amino acid that improved schizophrenic symptoms by acting on dopaminergic receptors (Suresh and Raju, 2013).

**FIGURE 5:**
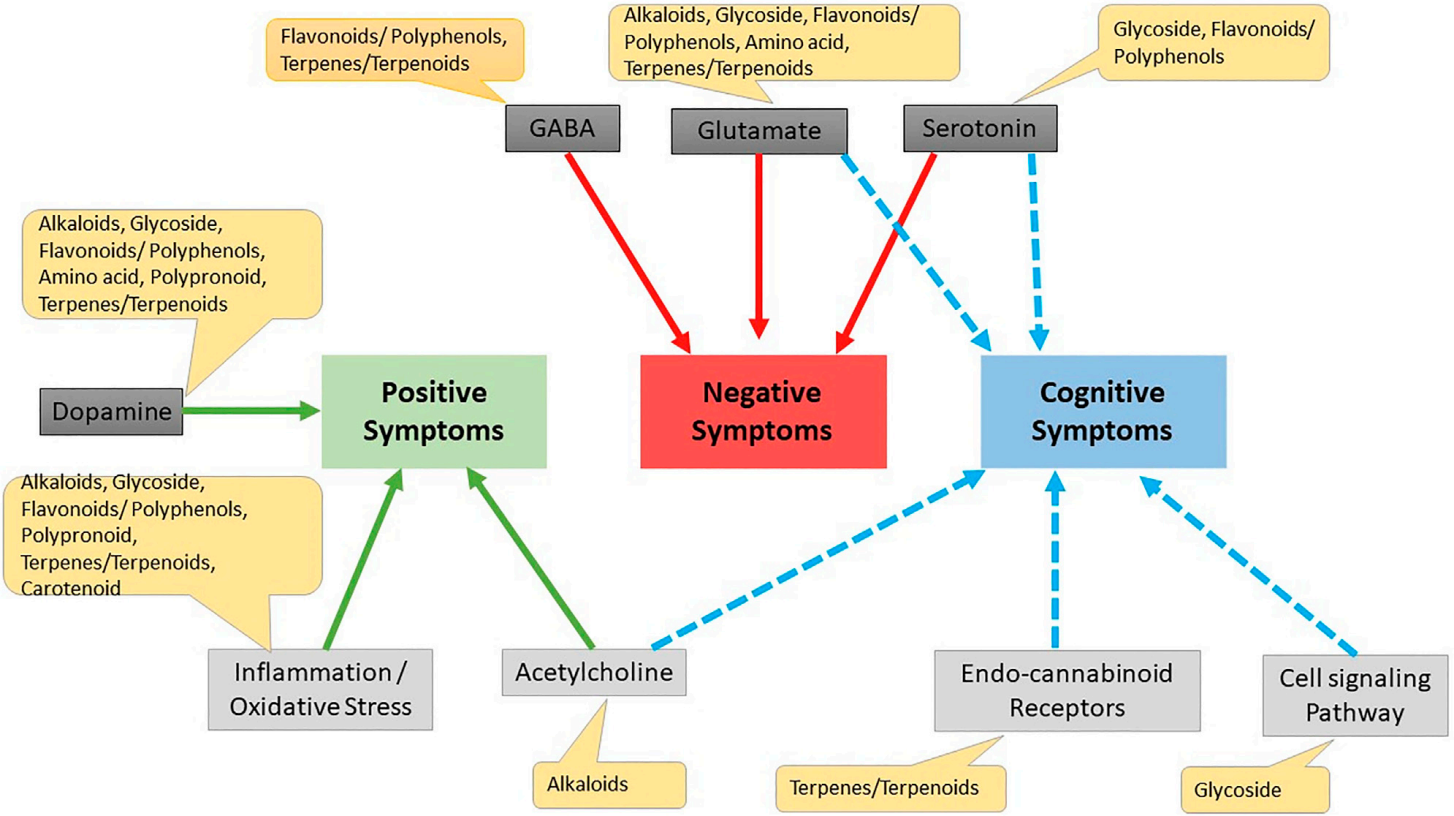
Effect of different phytochemicals on different drug targets for schizophrenia. Cell signaling pathway Erbβ cell signaling pathway, wnt/β-catenin Akt/GsK3β pathway.

Kava is a known herb for several brain disorders and its activity was reported due to its constituent kavapyrone. Kavapyrone is a potential candidate for treating schizophrenia as it increases GABA-A receptor density and blocks glutamate release (Kumari et al., 2011). Withaferin A, Withanolide A, Withanolide B and Withanolide D are steroidal lactones which have shown positive effects on NMDA receptors through docking studies and can be useful in schizophrenia after further evaluation (Kumar and Patnaik, 2016). The effect of various phytochemicals on positive, negative and cognitive symptoms is summarized in Figure 5.

## Conclusion

Schizophrenia is a multifactorial disease of complex etiology and pathogenesis that necessitates multiple targeted drug candidates for the improvement of positive and negative symptoms, and cognitive impairment. Natural drugs such as phytochemicals have demonstrated the therapeutic potential in the management of schizophrenia through modulation of oxidative stress, neuro-inflammation, immune system alterations and downstream signaling pathways, which are the hallmarks of disease. Alkaloids, glycosides, terpenes, terpenoids, polyphenols, flavonoids, poly-propanoids, steroidal lactones and amino acids are among the major classes of phytochemicals that have shown anti-schizophrenic activity in preclinical investigations. Apomorphine, luteolin, apigenin, caryophyllene, cannabinoids, baicalin and reserpine are among the phytochemicals that have demonstrated the anti-schizophrenic potential in human studies.

Therefore, it is reasonable to propose that the phytochemicals might be promising candidates for developing new agents with protective and therapeutic benefits against schizophrenia. Moreover, additional preclinical and clinical research is required for establishing pharmacokinetic and toxicity studies of phytochemicals, and their best possible combinations to minimize undesirable adverse effects. Unfortunately, in spite of abundant neuroprotective potential of the phytochemicals against schizophrenia, long-term studies of these agents against schizophrenia have not been carried out to address the effects of these agents to retard the progression of disease. Furthermore, the exact doses and combinations of phytochemicals should be investigated in clinical research to demonstrate the efficacy and safety in schizophrenic patients.

## Abbreviations

5HT, 5 Hydroxy Tryptamine; Akt, Protein Kinase C; BDNF, Brain-derived neurotrophic factor; COMT, Catechol-O-methyl transferase; DAOAG72, d-amino acid oxidase; DISC1, Disrupted in schizophrenia; DNA, Deoxyribonucleic acid; DTNBP1, Dysbindin; EPS, Extra pyramidal symptoms; ERK, Extracellular signal-regulated kinases; GABA, Gamma amino butyric acid; GSK 3, Glycogen synthase kinase-3; IL, Interleukin; NRG1, Neuregulin 1; nAChR-Nicotinic acetylcholine receptors; NMDA, N-methyl-d-aspartate; PDE, Phosphodiesterase; PRODH, Proline dehydrogenase; RNS, Reactive nitrogen species; ROS, Reactive oxygen species; RGSR, Regulator of G protein signaling 4.
